# Signals from the interface: protein nanoclusters stabilize biomolecular condensates

**DOI:** 10.1038/s41392-022-00876-5

**Published:** 2022-01-13

**Authors:** Christian Hoffmann, Branislava Rankovic, Dragomir Milovanovic

**Affiliations:** Laboratory of Molecular Neuroscience, German Center for Neurodegenerative Diseases (DZNE), 10117 Berlin, Germany

**Keywords:** Cell biology, Biochemistry, Developmental biology

In a recent study, Folkmann and colleagues^1^ show that protein nanoclusters at the surface of biomolecular condensates act as Pickering agents stabilizing these emulsions in cells.

The accumulating evidence suggests that cytosol represents a complex emulsion where multiple membrane-bound organelles and biomolecular condensates co-exist together.^2^ Each of these condensates is a crucible that can enrich for numerous proteins, RNAs, and even the entire membrane-bound organelles, such as the cluster of synaptic vesicles or the Golgi stacks. The molecules in these condensates remain highly mobile, readily exchange with the surrounding environment and grow/coarsening over time (by coalescence or Ostwald ripening), often towards the round structures to minimize the surface tension.

The fluid nature of condensates suggests that molecules maintain high diffusion within these condensates and readily exchange between the condensate and the surrounding medium. However, data across many studies show that these condensates can evolve into less dynamic states where molecules binding within condensates increases over time, forming so-called aging Maxwell fluids.^3^ Failure to regulate this maturation process often leads to insoluble aggregates, a characteristic of many neurodegenerative diseases and cancers. For example, some biomolecular condensates in cells—such as P granules in developing *C. elegans* embryo—are shown to have fluid-like properties but refrain from coalescing into a single large condensate despite the timescale of hours or even days.^4^ Several key questions emerge as to how nature evolved to regulate the stability of multiple condensates and yet prevent their maturation into insoluble aggregates.

Recently, the lab of Geraldine Seydoux discovered that nanoscale protein clusters can act as Pickering agents and stabilize P granules, a class of RNA-containing granules in the germline.^1^ Pickering emulsion is stabilized by solid particles that adsorb on the interface of two phases, often used in material science,^5^ drug, and food industry.^6^ P granules show remarkable dynamics during oocyte maturation and zygote polarization with rounds of dissolution and assembly, as well as the spatial patterning within the zygote. P granules are heterotypic condensates assembled by PGL proteins (PGL-1 and PGL-3) and regulated by disordered proteins MEGs (MEG-3 and MEG-4), MBK-2 (DYRK3 kinase in mammals), and MEX-5, an RNA-binding protein.

**Fig. 1:**
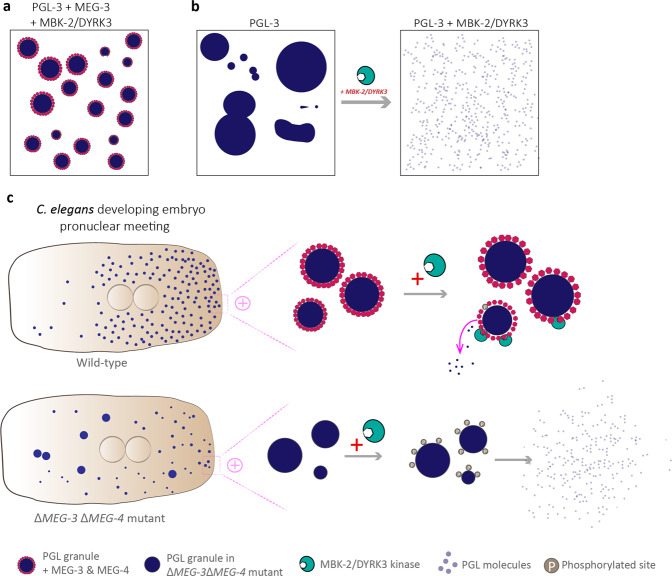
MEG-3 nanoclusters as Pickering agents stabilizing PGL granules. **a** MEG-3 nanoclusters assemble at the surface of PGL-3 condensates and prevent them from coarsening. **b** In the absence of MEG-3, MBK-2/DYRK3 kinase dissolves PGL condensates. **c** MEG-3 nanoclusters act as molecular beacons in developing embryos of *C. elegans* (top), recruiting MBK-2/DYRK3 kinase, thereby allowing for PGL diffusion in and out of granules. In the absence of MEG-3 stabilization (bottom), MBK-2/DYRK3 completely dissolves PGL-3 condensates

While PGL-3 alone is coarsening into larger droplets in vitro, the addition of MEG-3 prevents this process resulting in PGL-3 condensates being stable over hours, resembling their stability observed in *C. elegans* zygote (Fig. 1a, b). To quantify the effect of MEG-3, the authors turn to passive microrheology and imaging coalescence of condensates over time. Indeed, MEG-3 reduces the surface tension of PGL-3/RNA condensates four times while viscosity remains unaltered. This aligns well with the concept of Pickering emulsions, as when clay particles stabilize oil droplets in water.^5^ Indeed, the in vivo comparison of P granules (granule volume and PGL concentration) in wild-type and animals lacking MEG-3 and MEG-4, suggests that MEGs stabilize many small PGL-3 emulsions during oocyte-to-zygote transition.

Symmetry breaking and transitioning of the zygote from unpolarized to polarized is tightly accompanied by the formation of cytosolic gradients of RNAs and protein factors. PGL-3 condensates are considered to accumulate at the posterior side in response to MEX-5-driven RNA accumulation at the anterior of the zygote inducing the saturation gradient. However, Folkmann and colleagues experimentally show that MEX-5 alone is not sufficient for this patterning and that MEGs are essential for positioning and accumulation of PGL-3 condensates at the posterior of the zygote. MEG nanoclusters at the surface of PGL-3 condensates act as molecular beacons recruiting MBK-2/DYRK3 kinase that is required for P granule dissolution during polarization (Fig. 1c).

The experimental observations of P granule dynamics in oocytes and during polarization of zygote were used to build a theoretical model, which took into account three important elements. In the model, the conversion rate of soluble PGL-3 molecules to PGL-3 molecules engaged in the condensates depends on the availability of MEG nanoclusters and recruited MBK-2/DYRK3 kinase. The heterogenous distribution of RNAs (i.e., through MEX-5 as an RNA-binding protein) affects the positioning of PGL-3 condensates. Finally, the differences in chemical potential at the condensates in the posterior end of the zygote depend on the asymmetry of the MEGs, as necessary surface stabilizers. Indeed, such a theoretical framework recapitulated the dual nature of MEGs at supersaturated concentrations of PGL-3 molecules: they both act as Pickering reagents stabilizing the condensate and are necessary to recruit MBK-2 kinase that solubilizes PGL-3 granules, thus allowing for their growth and recruitment of new molecules.

The discovery that biomolecular nanoclusters adsorbed at the surface of condensates can act as Pickering agents goes beyond P granules in *C. elegans* oocyte development. This concept puts forward several testable hypotheses that protein nanoclusters that accumulate at the interface of biomolecular condensates can stabilize the condensates across different time scales and act as molecular beacons for recruitment of enzymes able to couple the metabolic state of the cell with the condensate dis/assembly.
